# Globular and Fibrous Proteins Modified with Deep Eutectic Solvents: Materials for Drug Delivery

**DOI:** 10.3390/molecules24193583

**Published:** 2019-10-04

**Authors:** Wanwan Qu, Riina Häkkinen, Jack Allen, Carmine D’Agostino, Andrew P. Abbott

**Affiliations:** 1 Department of Chemistry, University of Leicester, University Road, Leicester LE1 7RH, UK; wq10@le.ac.uk (W.Q.); ja462@le.ac.uk (J.A.); 2 VTT Technical Research Centre of Finland Ltd., Tietotie 4 E, FI-02150 VTT, Finland; Riina.Hakkinen@vtt.fi; 3 School of Chemical Engineering and Analytical Science, University of Manchester, Oxford Rd, Manchester, M13 9PL, UK; carmine.dagostino@manchester.ac.uk

**Keywords:** zein, soy, gelatin, deep eutectic solvent, drug delivery, transdermal

## Abstract

Proteinaceous materials have numerous structures, many of which aid in the roles they perform. Some need to impart strength while others need elasticity or toughness. This study is the first to investigate the modification of both globular and fibrous protein, namely, zein, soy protein and gelatin, using deep eutectic solvents (DES) to form bioplastics, which may have application in drug delivery systems. The effects of DES content on the thermal and mechanical properties of the material were determined. Zein and soy are globular proteins, which both showed a significant change in the properties by the addition of DES. Both of these materials were, however, weaker and less ductile than the starch based materials previously reported in the literature. The material made from gelatin, a fibrous protein, showed variable properties depending on how long they were in contact with each other before pressing. Conductivity and NMR measurements indicate the existence of a continuous liquid phase, which are useful in the demonstrated application of transdermal drug delivery systems. It is shown that pharmaceutical DESs can be gelled with gelatin and this method is three times faster at delivering a pharmaceutical active ingredient across the skin barrier than from a corresponding solid formulation.

## 1. Introduction

Plastics based on natural polymers are frequently studied, as they are water-soluble, biocompatible, biodegradable and non-toxic. Furthermore, many natural animal proteins such as keratin, collagen, elastin, and silk are relatively cost-effective and sustainable. They are easily derived from their natural sources and simple to process under mild conditions. In their pure, dry state most proteins are crystalline and brittle but the addition of modifiers can change the properties significantly. Several products based on proteins such as soy, whey and casein are available and numerous more have been tested for food, packaging and biomedical applications [1,2,3].

Polysaccharides, particularly starch, can also be plasticized using small polar molecules such as glycerol or urea. These are completely compostable and are becoming popular for short-life packaging applications. The polysaccharides can slowly recrystallize which aids their decomposition but can compromise their shelf life. It has previously been shown that Deep Eutectic Solvents (DES), which are mixtures of quaternary ammonium salts and hydrogen bond donors [4], can plasticize starches by breaking up the starch hydrogen bonding structure and enabling movement of amylose and amylopectin chains past each other [5]. The presence of the DES prevents starch recrystallization and it has been found that samples retain mechanical properties 5 years after production if moisture is prevented from entering the material [5,6].

Fundamentally DES-modified starches and DES-modified proteins should behave in the same way, i.e., a proportion of the DES will strongly bind to the polymer chain whereas the remainder will be less strongly bound and act as a lubricant/plasticizer. However, the properties of the materials may be expected to be different as the proteins will be more hydrophobic and the intermolecular interactions are between different functionalities, not just amides. In starch the interactions are all between -OH functionalities. Another fundamental difference between starches and proteins is that the former has repeating monomer units of the same monomer whereas the latter has a more random sequence of monomers and the secondary structure is more ordered. Consequently, zein has been reported to be insoluble in many choline chloride (ChCl)-based DESs, whereas starch and gelatin are soluble up to 40 wt% [7,8]. Thermoplastic starches will always have elastomeric properties due to the coiled structure of amylopectin whereas the thermoplastic proteins might behave differently depending on whether they are predominantly α- helices or β-sheets. Previously it has been shown that the secondary structure of the proteins is retained while their tertiary structure is disrupted in DESs [9,10]. However, the proteins denaturize irreversibly above 80 °C, indicating that the DES does not improve the thermostability of the proteins. The partially unfolded structure of the protein in DESs has been reported to decrease the protein activity, but after removal of the DES, the protein activity is completely recovered [9].

There are few studies on DES-modified bioplastics from proteins with various outcomes. Leroy et al. plasticized zein with 30 wt% of urea/ChCl and glycerol/ChCl DESs by thermomolding. Brittle materials were obtained, most probably due to the poor solubility of zein in these DESs. However, thermoplastic starch/zein (90:10) blends were successfully made with good mechanical properties. A study by Qin et al. [7] showed that 22 wt% of gelatin mixed with ethylene glycol/ChCl DES resulted highly stretchable, conductive gel suitable for ionic skin applications.

It has recently been shown, that deep eutectic formulations can be used to liquefy active pharmaceutical ingredients, which can circumvent issues associated with polymorphic drugs [11]. The aim of this study is to investigate the properties of DESs when mixed with three proteins; zein, soy and gelatin to try to develop a material which can be used to deliver pharmaceutical DESs transdermally. The aim is to find a strong but flexible material and to ensure that the DES remains as continuous, microscopic pools within the material.

## 2. Results and Discussion

### 2.1. Plasticisation of Zein

Zein is a protein present in maize seeds and plastics based on zein have been tested for food packaging applications using plasticizing agents to overcome its brittleness caused by extensive intermolecular forces. Zein contains a relatively high proportion of hydrophobic amino acids (leucine, proline and alanine). If both zein and starch can be processed in the presence of DES as plasticizers, the possibility of combining thermoplastic starch and plasticized zein will have great potential in the field of biodegradable plastics. Additionally, the low solubility of zein in water could increase the hydrophobic character of the corresponding thermoplastic starch compositions. The starch/zein (90:10) blends by Leroy et al. were promising, although the blended zein content was quite low [12].

Attempts have been made by multiple groups using different plasticizers, such as polyols and fatty acids [13]. The properties of the films vary considerably with the type and amount of plasticizer, the extrusion conditions and the conditioning settings. The ultimate tensile strength, UTS, can range from 0.4 to 23 MPa. Slightly higher strengths can be obtained with large mass fractions of PEG but these can be attributed to the properties of the modifier rather than the zein. The elongation at break ranges from 0 to > 200% and is inversely proportional to the UTS which is seen for most materials [8].

Table 1 shows the UTS and elongation at break for zein protein plasticised with Glyceline a mixture of 1 mol eq. ChCl and 2 glycerol. As expected, the UTS of the plasticised zein is less than that of the unplasticized material but the elongation at break is not significantly affected. This is unlike the behavior of starch with the same DES, which became significantly more ductile. The starch samples were also significantly stronger (UTS = 24 MPa) than zein once the extrusion and pressing conditions were optimized. In a like for like experiment without extrusion the strength of DES modified starch was only 5–6 MPa, i.e., comparable to the values in Table 1.

The low values for elongation at break suggest that the DES does not penetrate well into the globular structure of the protein and acts more like a lubricant than a plasticizer. A similar behavior was observed for DESs added to high-density polyethylene (HDPE) where interaction with the chain was minimal and the DES showed solubility < 3 wt% and this had negligible effect on the glass transition temperature or the UTS [14]. The inability of the DES to penetrate into the zein structure is possibly because the protein is relatively hydrophobic and may explain why the fatty acids and PEG acts as better plasticizers for zein than the polyols [8].

**Figure 1:**
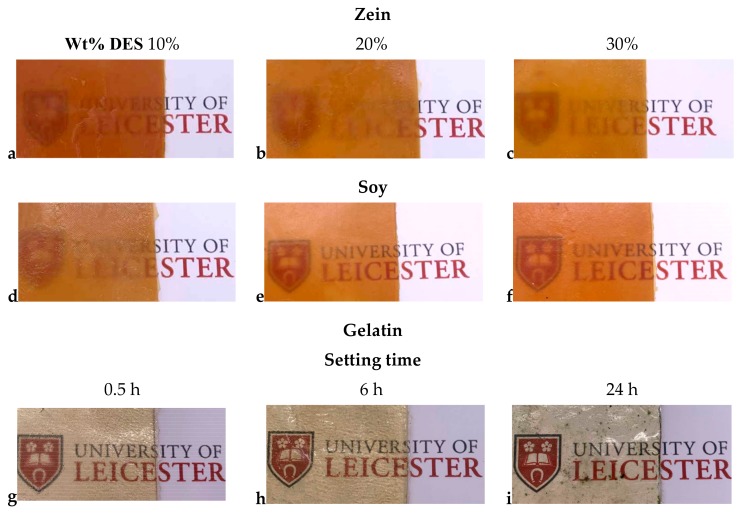
Photographs of zein (**a**–**c**) and soy (**d**–**f**) proteins with different DES contents and for gelatin samples with 20% DES after setting time of (**g**) 0.5, (**h**) 6 h, and (**i**) 24 h**.**

The ability of the DES to plasticize the zein protein can also be seen from Figure 1, which shows photographs of sheets with different DES content. It can be seen that all the sheets are relatively opaque showing that there are crystalline regions, which are at least as large as the wavelength of visible light which are causing significant scattering by the 1 mm thick sample.

**Figure 2:**
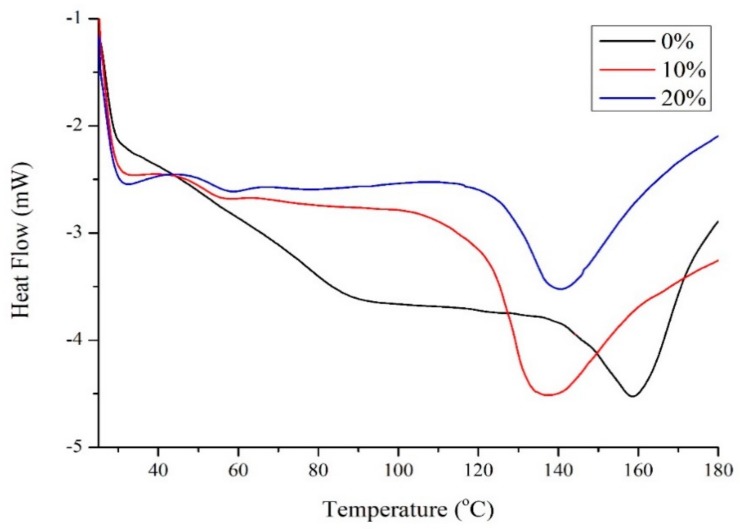
Transition temperatures for zein samples with 0%, 10% and 20% Glyceline measured by DSC.

Figure 2 shows the differential scanning calorimetry (DSC) results for zein with different amounts of Glyceline. Unmodified zein was found to have a *Tg* of 159 °C which is similar to the literature value of 162–165 °C [15]. The addition of 10 wt% Glyceline decreased the *Tg* value to 139 °C. The *Tg* for unmodified starch depends on water and amylose:amylopectin content but is generally in the range 50–60 °C. The addition of 10 wt% Glyceline to starch caused the *Tg* to drop to 13 from 22 °C, i.e., a similar magnitude to that observed for zein [6]. Higher plasticizer content did not significantly affect *Tg* for either zein or starch. The same effect has also been observed for polar modifiers with zein [16,17].

The phenomena that plasticizers like glycerol can act as both a plasticizer and an anti-plasticizer have been shown in food applications in recent years [18]. It has been reported that glycerol can exert different effects depending on its concentration. For example, Lourdin and colleagues reported that glycerol at a content below 12 wt% could increase the ductility of potato starch film while the ductility decreased when the amount of glycerol exceeded 12% [19]. At low content, Glyceline is suspected to decrease the local dipolar relaxation of the amorphous zein matrix and thus appear to act as an anti-plasticizer on the molecular level [20].

### 2.2. Plasticization of Soy Protein with Glyceline

Soy-based bioplastics have also been extensively studied using a variety of polyol based plasticizers [21,22]. They can absorb significantly more plasticizer than zein and this has led to their use as super-absorbers [23]. Table 2 shows the UTS and elongation at break data for soy protein modified with different amounts of Glyceline. It can be seen that more DES can be incorporated into soy protein compared to zein. The UTS values are lower than the corresponding zein materials but they are far less brittle and the elongation at break increases when 20 wt% DES is added. The same trend is observed for soy protein modified with glycerol where the UTS is in the region 7–40 MPa depending on content and processing conditions [24].

**Figure 3:**
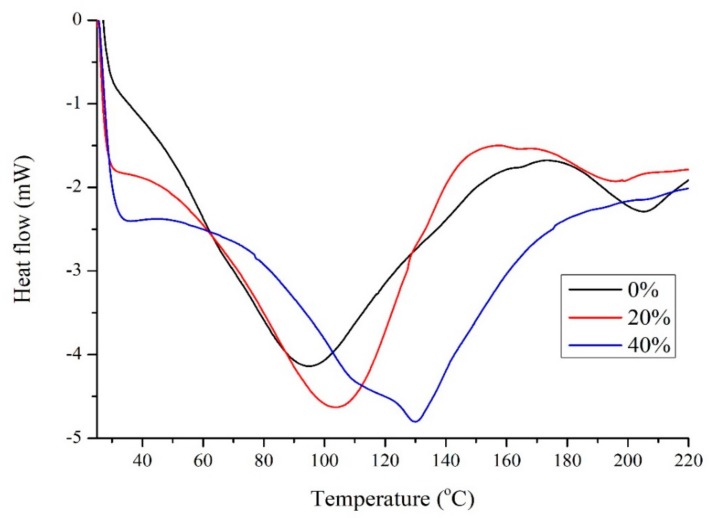
DSC for soy samples with 0%, 20%, and 40% Glyceline.

Figure 3 shows the DSC results for soy with different amounts of Glyceline. In the absence of DES two minima are observed at 95 and 206 °C which are characteristic denaturation peaks for 7S and 11S globulins which involves unfolding and breaking of hydrogen bonds, hydrophobic interactions and salt linkages at elevated temperature as well as decomposition [25]. The addition of Glyceline increased the temperature at which the first minimum occurs suggesting that DESs stabilize the 7S globulin while the 11S globulin is destabilized. There is no clear and obvious glass transition temperature in Figure 3 but this is common with soy protein. This supports the observation that proteins are partially unfolded in DESs [9,10]. The protein attempts to minimize its free energy by burying as many hydrophobic groups inside the material while exposing as many polar groups as possible to the plasticizer, which greatly weakens the short-range interaction and hence protein folding structures to change the property of the native protein.

The results above show that globular proteins such as soy and zein can be plasticized by Glyceline but the resulting material are generally weaker and more brittle than materials reported with PEG and fatty acid modifiers. Changes in plasticization effectiveness can be attributed to an increasing difficulty for the plasticizers to diffuse into the polymer matrix due to its hydrophobicity. This might result a phase separation, which might be advantageous in drug delivery systems. However, the poor physical properties of both modified proteins (weak, brittle and rigid) make them inappropriate for potential applications in drug delivery without further improvements.

### 2.3. Plasticization of Gelatin with Glyceline

Gelatin is a high molecular weight polypeptide composed of amino acids, mainly glycine, hydroxyproline and proline, making it more hydrophilic than the globular proteins used above. Like zein and soy, pure gelatin is brittle but can be plasticized by polyols to improve the mechanical properties and enable films to be produced. Fakhoury et al., showed that adding 10 wt% glycerol produced a material with a UTS of 108.28 ± 6.38 MPa while the addition of 20% modifier, caused it to decrease to 1.75 MPa [26]. The blend of different plasticizers has also been used to improve the functional properties of gelatin e.g., sorbitol [27] and polyethylene glycol [28] mixtures with glycerol both showed increased tensile strength compared to pure glycerol. As a small hydrophilic molecule, glycerol can be inserted between protein chains, increasing the distance between the protein chains and reducing their direct interactions, hence acting as a plasticizer interspaced in a protein network.

Singh et al. used aqueous solutions of ionic liquids (IL) like [C_8_mim]Cl and [C_4_mim][C_8_OSO_3_] to modify gelatin [29]. Results show that initially, the IL monomers interact with gelatin at the interface but at higher concentrations hydrophobic micro domains were observed. A study by Alaysuy investigated the effect of DESs on collagen in leather [30]. Results showed that the DES did not denature the collagen structure but was absorbed into the macroscopic pores and could be forced out of the structure with applied pressure, i.e., the DES did not change the crystalline structure of the collagen.

When Glyceline was used to modify gelatin a significantly different material was produced than with zein or soy proteins, as shown in Figure 1. Interestingly, it was also discovered that the mechanical properties of DES modified gelatin is strongly influenced by the pre-mixing time of the mixture before pressure-heat treatment. This was not observed with either zein or soy protein. Table 3 shows that if the gelatin-Glyceline (20 wt%) mixture was pressed and heated at 75 °C immediately after mixing, then a strong but brittle material is obtained. If the mixture is stored in a sealed container for 24 h before pressing then a weaker but more flexible sheet is produced.

The dependency of the mechanical strength on the mixing time shows that the infusion of the DES into the gelatin structure is relatively slow due to the high viscosity of the DES and the slow movement of the polymer chains. The same observation was noted for the modification of collagen with DESs [31]. The properties of the materials remained the same after pressing, i.e., they showed no time-dependence, showing that the pressing process does not further promote the penetration of DES into the material. These results suggest that the DES interrupts protein–protein interactions and causes an increase in chain mobility in gelatin. Moreover, the elongation at break of the 24 h sample is very high, which can suggest highly entangled protein chains caused by dissolution. Similar correlation between dissolving plasticizer and elongation at break was observed in starch-based materials [32]. The protein:DES composition of 80:20 wt% was chosen for follow up experiments as it made samples with the most plasticizing effect without DES leakage.

**Figure 4:**
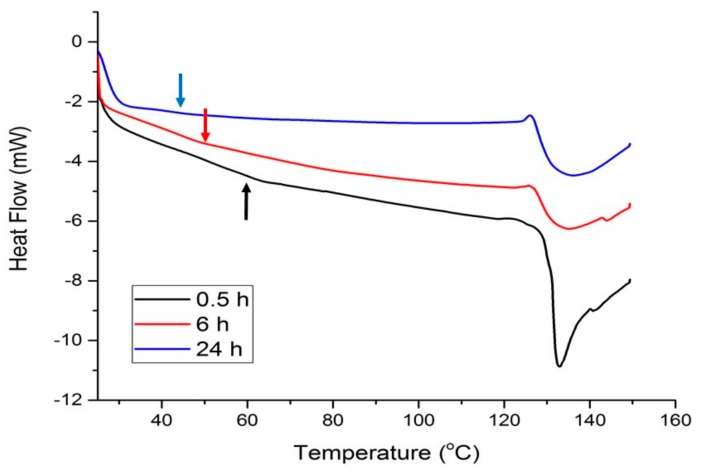
DSC curves for gelatin samples with 20% Glyceline after different setting times.

Figure 4 shows the thermal response of materials modified with 20 wt% Glyceline as a function of setting time and it can be seen that as the time increases the point of inflection, corresponding to *T_g_* decreases from 60 to 40 °C. It can be observed that the peak shape changes to less sharp during time (from 0.5 h to 6 h). This most probably indicates that the protein structure changes from crystalline to more amorphous as the DES infuses deeper into the structure. The resulting *T_g_* value after 24 h is comparable with the reported value for the glycerol plasticized films made from bovine-hide gelatin which was 41.5 °C [33].

Figure 1 shows optical photographs of samples pressed under identical conditions to create 100 × 100 × 1 mm sheets. It can be seen that the samples change color from straw colored to almost colorless. The color does not come from a chromophore as the components are effectively colorless and so it probably originates from light scattering due to the crystallinity of the material as a crystalline material will scatter light due to the size of the crystallites whereas an amorphous material will be more transparent.

**Figure 5:**
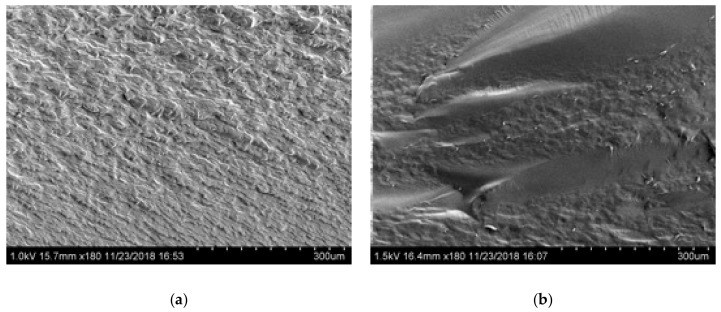
Morphology of gelatin samples after different setting time (**a**) 0.5 h and (**b**) 24 h.

**Figure 6:**
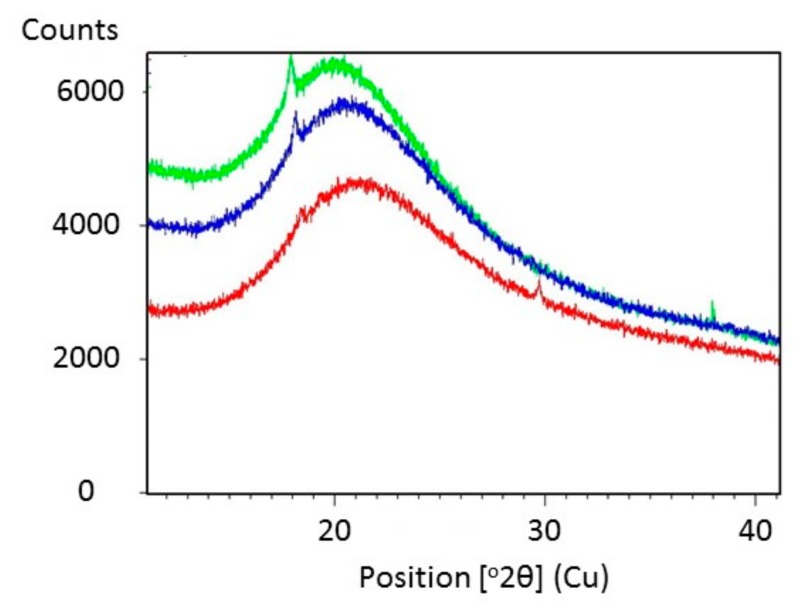
X-ray diffraction pattern of gelatin with 20% Glyceline after different setting time, (green) 0.5 h (blue) 12 h (red) 24 h.

Figure 5 shows SEM images of Glyceline modified gelatin as a function of setting time. As suspected, the SEM images indicate that the sample pressed immediately after mixing has a crystalline structure whereas that made after 24 h setting time is more amorphous. This is confirmed by the XRD data in Figure 6 where a sharp peak at 18° is observed for the sample made immediately and largely absent from the sample after 24 h.

**Figure 7:**
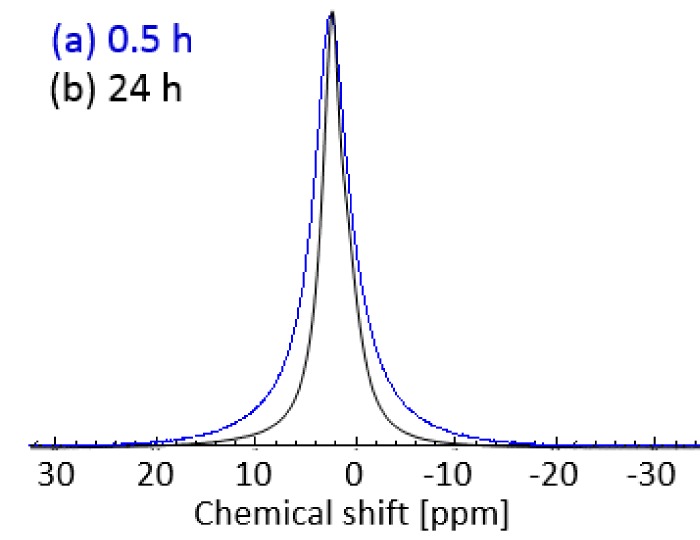
^1^ H-NMR spectra of gelatin sample with 20% DES prepared using a setting time of (**a**) 0.5 h and (**b**) 24 h at 21 °C.

To investigate the phase behavior and dynamics of Glyceline ^1^H NMR spectra as well as *T*_1_ and *T*_2_ relaxation measurements were carried out on the samples pressed after setting times of 0.5 and 24 h. ^1^H NMR spectra of gelatin samples with 20% Glyceline are shown in Figure 7. Figure 7a shows the spectrum of the gelatin sample prepared using a setting time of 0.5 h, whereas Figure 7b shows that of the same sample prepared using a setting time of 24 h.

A clear difference between the spectra can be seen in the broadness of the NMR peak. In particular, the peak of the sample at 0.5 h setting time (Figure 7a, full width at half maximum, FWHM, of the peak of 4.3 ppm) is much broader compared to that of the sample with 24 h setting time (Figure 7b, FWHM of the peak of 2.8 ppm). This clearly suggested a more restricted spin molecular mobility for gelatin samples prepared using short setting times as opposed to longer setting times, the sample with 30 min of setting time has the chordal modulus of 2270 Nm^−2^ while the 24 h sample was 0.95 Nm^−2^. The results ties in well also with the physical appearance of those samples, with the 0.5 h setting time sample being relatively much rigid compared to the more flexible 24 h setting time sample.

**Figure 8:**
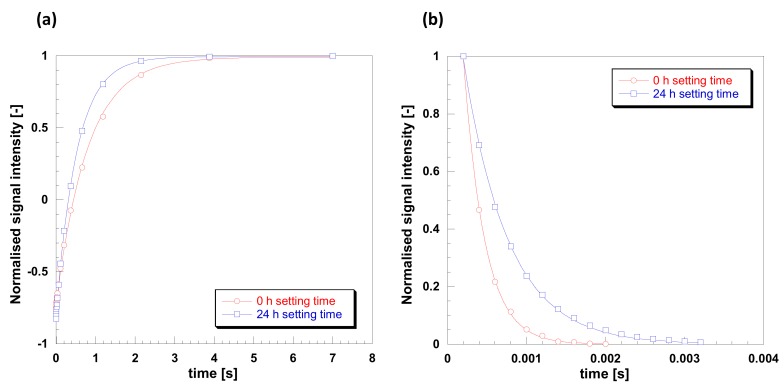
Plots for (**a**) T_1_ and (**b**) T_2_ relaxation for gelatin with 20% Glyceline for setting times of 0.5 and 24 h setting times at 21 °C.

In order to further elucidate spin molecular dynamics in such samples, *T*_1_ and *T*_2_ NMR relaxation measurements were carried out. Such measurements can give a clear indication of molecular mobility when analyzed against the Bloembergen-Purcell-Pound (BPP) theory of relaxation [34]. According to the theory, mobile phases, such as non-viscous fluids, tend to have high values of relaxation times, in the order of seconds, with *T*_1_ being essentially the same as *T*_2_. This region is also called fast-tumbling motion regime. As mobility becomes more restricted, the two relaxation times diverge, with *T*_1_ > *T*_2_; in particular *T*_1_ decreases reaching a minimum and then increases again, whereas *T*_2_ keeps decreasing and for solid samples its values may go as low as microseconds. This behavior is typical of the slow-motion regime, mostly observed for solids. *T*_1_ and *T*_2_ plots for the gelatin sample studied here are shown in Figure 8. Data for *T*_1_ were fitted using Equation (1), shown in Figure 8a, whereas data for *T*_2_ were fitted using Equation (2), shown in Figure 8 b. The quality of the data is excellent and from such fittings is possible to calculate the values of relaxation times. For the 0.5 h sample we have *T*_1_ = 800 ms and *T*_2_ = 0.27 ms, whereas for the 24 h setting time we have *T*_1_ = 522 ms and *T*_2_ = 0.56 ms. This increase in *T*_1_ and further decrease in *T*_2_ for the 0.5 h setting time sample compared to the 24 h setting time sample clearly indicates a significantly lower molecular dynamics and that we are in the slow-tumbling motion regime of the BPP theory [31].

The results of the ^1^H NMR spectra and *T*_1_ relaxation time agrees with those shown in SEM and XRD data and indicates that for a 0.5 h setting time a more ordered and rigid structure with a crystal structure is being formed, whereas for a 24 h setting time the structure becomes totally amorphous and more mobile. This shows that dissolution of the crystalline regions with a relatively viscous liquid is slow at ambient temperature.

To understand the mobility of ions in more depth the sheet conductivity was measured using a 4 point conductivity probe. It was found that the conductivity on the sample after a 0.5 h mixing time was 1.4 (± 0.64) × 10^−6^ Sm^−1^. After 6 h setting time the conductivity was 5.9 (± 1.8) × 10^−5^ Sm^−1^ and this rose to 1.7 (± 0.18) × 10^−4^ Sm^−1^ after 24 h. These values compare with a solution conductivity of 0.15 Sm^−1^ for pure Glyceline. The first point to note is that the films are conducting which means that the DES forms a continuous phase, i.e., it is not just a surface adsorbed layer. The conductivities are lower than that of pure Glyceline showing that the gelatin restricts the movement of ions as would be expected. The sample made after 24 h setting time is approximately 1000 times less conductive than the pure DES, which is due to the hindered mobility of the ions by the polymer matrix. Given that the XRD results indicate a mostly amorphous phase, the conductivity and NMR data suggest a network of DES held together with loosely packed gelatin coils. For samples which were pressed immediately after mixing, the conductivity is approximately two orders of magnitude smaller than the one after 24 h setting time. This shows that the crystalline regions are slow to solvate and hinder the flow of ions to an even greater extent. The DES is clearly held in a network of continuously linked pools and could act as a liquid delivery system. One application could be in the delivery of active pharmaceutical ingredients, APIs, particularly in a transdermal application method.

APIs can be formulated into DESs either if they contain a quaternary ammonium group (amines are often delivered as HCl salts to improve solubility) or if they have alcohol, amide or carboxylic acid moieties. These pharmaceutical DESs, PDESs, have been shown to enable API solubility even when the component itself is relatively insoluble in water [7]. The concept investigated here is whether PDESs can be used to modify gelatin and whether this material can be used to deliver APIs into the body transdermally. There are numerous methods available to test the efficacy of transdermal delivery systems and each has their own advantages and disadvantages [35]. In this case, a rehydrated leather sample was used to test the difference in absorbance rates of the pure API and an API in the form of a PDES.

**Figure 9:**
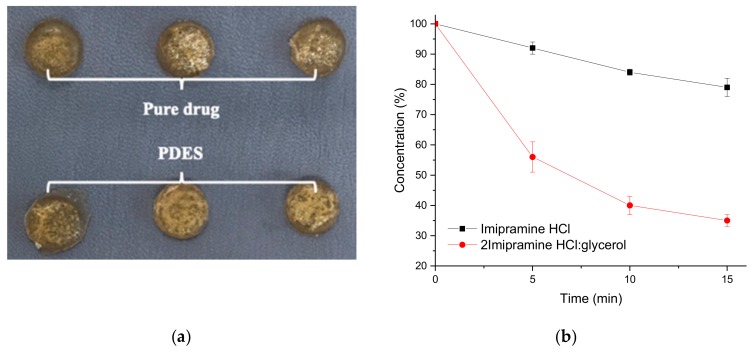
(**a**) Gelatin-based drug patches on saline wetted bovine hide for transdermal drug delivery testing. (**b**) Remaining PDES in patches after different amount of contact time with bovine hide with Imipramine HCl and 2 Imipramine HCl: Glycerol.

The human skin is made up predominantly of collagen and has a moisture content between 15 and 30 wt%. To mimic this, a chromium tanned bovine leather sample was soaked in saline solution for 30 min until a moisture level of 27 ± 0.5% was achieved (tested using a Testo 606-1 Moisture Meter). A PDES containing 2 Imipramine HCl: 1 glycerol was made by the method described previously [11]. Imipramine HCl is a tricyclic amine used as an anti-depressant. Two types of patches were made containing firstly 20 wt% pure Imipramine HCl in powder form in gelatin and secondly the same mass of Imipramine HCl in PDES formulation with glycerol in gelatin. The latter was pressed after a setting time of 12 h. The modified gelatin sheet had a UTS of 25 ± 2.5 MPa and an elongation at break of 2.5 ± 0.6%. These are similar to the data shown in Table 3 showing that the properties of the DES (in this case by changing choline chloride for Imipramine HCl) do not significantly affect the mechanical properties of the gelatin sheet. In each experiment, two drug patches were removed at 5 min intervals, one of each type, from the saline wetted hide (Figure 9a) and this experiment was repeated 3 times. The patches were dissolved in 500 mL of saline solution at 37 °C. After all the patches were fully dissolved, the resulted solutions were then analyzed by direct UV spectrophotometry to determine the remaining drug content. The collected data was calculated and plotted versus time (Figure 9b).

Figure 9b shows that the uptake of API into the saline wetted leather is significantly faster from the PDES formulation than from the solid powder API showing that the solubilization of the API is faster from the PDES as might be expected. Over the 15 min experiment three times more API is extracted from the PDES modified gelatin than from the solid API. This shows not only the efficacy of PDESs but also the suitability of DES modified gelatin as a method of trans-dermal drug delivery.

## 3. Material and Methods

Glyceline was prepared using choline chloride (ChCl) (Sigma-Aldrich, >98%, Gillingham, UK) and glycerol (Fischer Scientific, >98%, Loughborough, UK), which were mixed together in a 1:2 ratio respectively. The two components were stirred on a hotplate at 50 °C until a homogeneous fluid was formed. Protein samples were prepared by mixing the corresponding protein powder with the prepared Glyceline in different weight ratio. The resultant mixture was then thoroughly mixed manually without extruding. The mixture was then placed between two copper plates lined with non-stick silicone sheets and equipped with a 1 mm copper separating unit with a 100 mm^2^ aperture. The resultant sandwich was then placed in a hydraulic press (Fontune Grotnes Laboratory Press TH400, Chicago, IL, USA) and a force of 120 kN was applied to the sample at different temperature (zein 110 °C, soy 130 °C, gelatin 75 °C) for 10 min. The sample was subsequently cooled to ambient temperature whilst remaining under pressure prior to removal from the hydraulic press. The cooled sample was subjected to a mechanical ‘dog-bone’ press (Ceast Hollow Die Punch, Type 6051 from Instron, High Wycombe, UK) to cut into test shapes (test area size: L = 30 mm, W = 4 mm, D = 0.7–1.2 mm). The gelatin sample containing the pharmaceutical deep eutectic system was prepared in exactly the same way was the corresponding Glyceline sample. A mixture of 2 Imipramine HCl: 1 glycerol was heated to 50 °C and stirred until a homogeneous liquid was obtained. This liquid was then added to powdered gelatin (20 wt% liquid: 80 wt% gelatin) and left to stand for 12 h before pressing a 1 mm thick sheet at a force of 120 kN and 75 °C. Discs with a diameter of 1 cm, were pressed from the sheet and placed on a wetted saline hide for different lengths of time to determine the amount of Imipramine HCl extracted.

Tensile testing was then performed on the dog-bone samples using an Instron 3343 tensile apparatus (Instron Ltd., High Wycombe, UK) with a load cell of 500 N. The material stress and strain were controlled by Instron Bluehill 2 software (Instron, High Wycombe, UK) and average numerical values were taken from eight or more samples. In each case, the thickness of the sample was measured using a micrometer and subjected to a strain rate between 2–10 mm min^−1^.

Differential Scanning Calorimetry (DSC) analysis was then performed to analyze thermal events with the samples. A Mettler Toledo DSC1 was used to determine glass transition temperatures, *Tg* and the results analyzed with STARe software (Mettler Toledo, Leicester, UK). Samples (5–10 mg) were placed on in a standard aluminium DSC pan before the lid of the pan was pierced. The sample was placed within the furnace next to an, otherwise identical, empty reference pan. The samples were heated from 25 °C to an upper limit at a rate of 10 °C/ min then held at the upper limit for 5 min. The upper limits were Zein 200 °C, Soy 250 °C and Gelatin 150 °C.

Sheet conductivity was determined using an Ossila 4 point conductivity probe (Ossila, Sheffield, UK). X-ray diffraction was carried out using a powder diffractometer, Bruker D8 Advance that was equipped with a LynxEye linear position sensitive detector and a 90-position autosampler (Brucker, Durham, UK). The diffraction patterns were collected using the following conditions: 2θ range: 4°–60°, step size: 0.02°, step time: 15 s taken over 11.5 h.

For the gelatin with Glyceline samples, all the NMR experiments were performed in a Biospin DMX 300 (Bruker, Durham, UK) operating at a ^1^H frequency of 300.13 MHz. NMR nuclear spin relaxation times *T*_1_ and *T*_2_ were measured using standard inversion recovery [36] and CPMG [37] techniques, respectively. Experimental data were fitted using single exponential functions. The *T*_1_ relaxation time constant was obtained by fitting the experimental data to the equation:

(1) $$S\left(t\right)=S_{0}\left[1-2\exp\left(-\frac{t}{T_{1}}\right)\right]$$

The T2 relaxation time constant was obtained by fitting the experimental data to the equation:

(2) $$S\left(t\right)=S_{0}\exp\left(-\frac{t}{T_{2}}\right)$$

In Equations (1) and (2) *S*(*t*) represents the NMR signal intensity as a function of the time *t*. The measurements were carried out at 21 °C. A Bruker Variable Temperature unit, BVT 3000 (Bruker, Durham, UK), was used to set the required temperature for each experiment. The sample species was placed in a 5 mm NMR tube, then loaded into the NMR probe and left for 20 min at the desired temperature in order to reach thermal equilibrium

## 4. Conclusions

This study has assessed the idea of using both globular and fibrous protein in DES blends to form bioplastics which may have application in drug delivery systems. The effect of DES content on the thermal/mechanical properties of the material was determined. In each case, a homogeneous material was produced. However, the zein-based material was weak and brittle and of very little mechanical integrity. The material formed from soy protein was slightly stronger and has a larger elongation, particularly when 20 wt% Glyceline was added. Both of these materials were, however, weaker and less ductile than the starch-based materials previously reported in the literature. When gelatin was plasticized by DES a very different material was obtained. Initially a very strong material was produced which displayed a strength similar to HDPE or polypropylene. Enabling the DES to contact with gelatin for different periods of time resulted in materials with different properties. After 24 h of contact followed by heated compression molding, a very flexible, transparent material was obtained. The longer the contact time, the more amorphous and more flexible the material became, as suggested also by NMR spectral and relaxation measurements. Pressure molded samples did not change their properties depending on how long they were stored. This shows that diffusion of the liquid into the gelatin was slow at ambient temperature and once pressed the gelatin retained its shape and the DES no longer diffused in the structure, i.e., the temperature fixed the gelatin structure. NMR and conductivity measurements showed that the DES was a continuous phase in the gelatin matrix and this enables the liquid phase to flow for drug delivery systems. This study has also shown the ability of gelatin modified with pharmaceutical DESs to deliver APIs across the skin barrier 4 times faster than using a solid API.

## Figures and Tables

**Table 1:** Tensile strength and elongation for zein samples with different Glyceline contents.

Glyceline Contents (wt%)	Ultimate Tensile Strength (MPa)	Elongation (%)	*T_s_* (°C)
0%	10.2 ± 1.4	1.38 ± 0.28	159
10%	6.5 ± 1.7	0.84 ± 0.09	139
20%	5.8 ± 0.78	0.80 ± 0.08	140

**Table 2:** Tensile strength and elongation for soy samples with different DES content.

Glyceline Contents (wt%)	UTS (MPa)	Elongation (%)
10%	2.0± 0.73	0.84 ± 0.28
20%	3.9 ± 0.74	17.2 ± 1.5
30%	2.1 ± 0.68	20.0 ± 4.6
40%	1.6 ± 0.55	18.6 ± 3.5
50%	0.37 ± 0.05	5.8 ± 1.8

**Table 3:** Tensile strength and elongation for gelatin samples with different DES composites with 0.5 h and 24 h setting times (30 min setting).

Glyceline Contents (wt%)	Ultimate Tensile Strength (MPa)	Elongation (%)	24 h Setting	Glyceline Contents (wt%)	Ultimate Tensile Strength (MPa)	Elongation (%)
10%	14.6 ± 2.8	3.5 ± 0.83		10%	0.84 ± 0.13	23.9 ± 6.8
20%	23.8 ± 5.7	3.3 ± 0.48		20%	0.05 ± 0.01	442± 80
30%	22.0 ± 6.0	22.6 ± 6.8		30%	0.01 ± 0.01	1265 ± 264
